# Amphetamine-Related Fatalities and Altered Brain Chemicals: A Preliminary Investigation Using the Comparative Toxicogenomic Database

**DOI:** 10.3390/molecules28124787

**Published:** 2023-06-15

**Authors:** Murad Tumayhi, David Banji, Ibrahim Khardali, Otilia J. F. Banji, Saeed Alshahrani, Saad S. Alqahtani, Safiah Muqri, Amal Abdullah, Wedad Sherwani, Ibraheem Attafi

**Affiliations:** 1 Department of Pharmacology & Toxicology, College of Pharmacy, Jazan University, Jazan 45142, Saudi Arabia; 2 Forensic Toxicology Services, Forensic Medical Center, Ministry of Health, Jazan 45142, Saudi Arabia; 3 Pharmacy Practice Research Unit, Department of Clinical Pharmacy, College of Pharmacy, Jazan University, Jazan 45142, Saudi Arabia; 4 Department of Clinical Pharmacy, College of Pharmacy, King Khalid University, Abha 61421, Saudi Arabia

**Keywords:** forensic toxicology, amphetamine-related fatalities, brain, omega-3 fatty acids, docosahexaenoic acid, Comparative Toxicogenomic Database (CTD)

## Abstract

Amphetamine is a psychostimulant drug with a high risk of toxicity and death when misused. Abuse of amphetamines is associated with an altered organic profile, which includes omega fatty acids. Low omega fatty acid levels are linked to mental disorders. Using the Comparative Toxicogenomic Database (CTD), we investigated the chemical profile of the brain in amphetamine-related fatalities and the possibility of neurotoxicity. We classified amphetamine cases as low (0–0.5 g/mL), medium (>0.5 to 1.5 g/mL), and high (>1.5 g/mL), based on amphetamine levels in brain samples. All three groups shared 1-octadecene, 1-tridecene, 2,4-di-tert-butylphenol, arachidonic acid (AA), docosahexaenoic acid (DHA), eicosane, and oleylamide. We identified chemical–disease associations using the CTD tools and predicted an association between DHA, AA and curated conditions like autistic disorder, disorders related to cocaine, Alzheimer’s disease, and cognitive dysfunction. An amphetamine challenge may cause neurotoxicity in the human brain due to a decrease in omega-3 fatty acids and an increase in oxidative products. Therefore, in cases of amphetamine toxicity, a supplement therapy may be needed to prevent omega-3 fatty acid deficiency.

## 1. Introduction

Amphetamine was first introduced as an over-the-counter medication and eventually restricted for specific conditions, such as narcolepsy and attention deficit hyperactivity disorder [1]. Globally, amphetamine and its analogs are widely misused psychostimulant drugs causing fatalities [2]. A study carried out in Saudi Arabia reported that amphetamine is the most misused drug after hashish [3]. Around 4–70% of Saudi patients undergoing addiction treatment were previously on amphetamine substance abuse [4]. Attafi et al. have reported an exponential rise in fatalities, from 18% in 2018 to 80% in 2020, due to the use of amphetamine along with other drugs in Jazan, Saudi Arabia [5]. Amphetamine abuse has found favor as it produces euphoria, increases performance, induces sexual arousal, and promotes weight loss [6]. Amphetamine directly impacts the central nervous system, increases the availability of monoamines, interferes with monoamine reuptake via monoamine transporters, and inhibits monoamine oxidase [7]. The primary site of action is the dopaminergic pathways in the mesolimbic system, particularly the reinforcement and reward circuitries [8]. Amphetamines increase extracellular dopamine concentration causing sustained stimulation of D1 and D2 receptors [9]. Amphetamine could also elicit its action on the corticostriatal system, affecting decision making, emotion regulation, reinforcement, and reward regulation [10,11].

Amphetamines are implicated in several adverse health outcomes such as intracerebral hemorrhage, cardiac arrest [2], psychosis [10], and suicide [12]. Additionally, indirect fatalities include traffic accidents and participation in high-risk behaviors with poor judgment. Specific cases of amphetamine-induced hyperthermia result in rhabdomyolysis [13] and multiple organ failure [14]. Moreover, the use of amphetamine can also result in associated problems such as HIV and hepatitis, both of which inflict a considerable burden on the health system [15]. An increase in amphetamine-dependent addicts among communities, hospitalization, rehabilitation, and costs contribute significantly to the global burden of disease.

Since it has been demonstrated that fatty acids (FAs) play an important role in neuronal survival, neurogenesis, and the regulation of brain inflammation, the altered FAs composition of the brain may be one of the causal factors in the central nervous system diseases associated with drug abuse [16]. Docosahexaenoic acid (DHA), an omega-3 fatty acid, has been found to be altered in postmortem analyses of the brains of individuals with schizophrenia, bipolar disorder, and major depressive disorders, when compared to normal brain tissue, implying a role for DHA in brain dysfunction [17,18,19,20]. Importantly, DHA, a primary FA constituent of the nervous system, promotes neuronal survival and neurogenesis, and has been found to reduce the induction of pro-inflammatory signaling proteins [21]. Thus, analyzing the DHA profile in postmortem brain tissues may reveal a biomarker linked to substances-related fatalities.

More research is needed to determine how amphetamine and its metabolites affect the brain, given the rise in mortality due to its use. Moreover, explicit judgments are difficult to make based on available data. Brain organic profiling is a reliable tool to illustrate alterations in organic components and related mental illnesses. Moreover, in our previous study, we discovered changes in the organic profile of the brain in amphetamine users [5]. Thus, we continued our investigation on the chemical–disease association based on the brain chemical profile of amphetamine in relation to the control and the occurrence of neurological disorders.

## 2. Results

**Figure 1:**
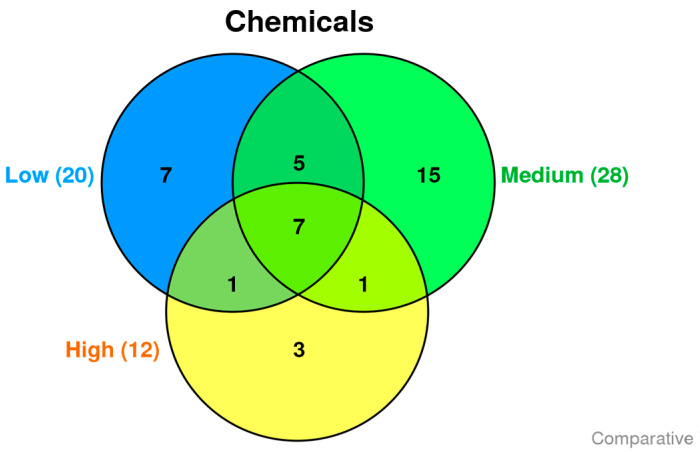
Chemical composition profile of brain samples from amphetamine-related fatalities. The blue, green, and yellow circles represent differentially detected chemicals in groups with low (Low), medium (Medium), and high (High) amphetamine concentrations, respectively, utilizing the CTD database.

Based on the amphetamine brain concentration, amphetamine related fatalities were subdivided into three groups (each group containing six cases). The three subdivided groups produced three distinct chemical sets with some overlap. The degree to which chemicals are detected similarly between these groups is represented by a Venn diagram (Figure 1). In the low, medium, and high groups, 251, 238, and 134 chemicals did not match any annunciated CTD chemicals, respectively.

In the low, medium, and high groups, 20, 28, and 12 chemicals matched the CTD database, respectively. The Venn diagram shows the three divided groups’ three distinct chemical sets with some overlap (Figure 1). The seven chemicals in common, including 1-octadecene, 1-tridecene, 2,4-di-tert-butylphenol, AA, DHA, eicosane, and oleylamide, are differentially detected in all three groups (Table 1).

These chemicals have Gene Ontology (GO) annotations that describe the biological processes and molecular functions associated with them. The CTD database tools were used to investigate the association between the seven common chemicals in all groups and diseases such as cardiovascular disease, metabolic disorders, nervous system disease, mental disorder, and cancers. Finally, our focus was on nervous system disease and mental disorder, so we chose CTD tools for further investigation. Among the chemicals common to all, only DHA, AA, and oleylamide were associated with curated diseases in the CTD database (Figure S1).

AA is commonly associated with 22 diseases, including cardiovascular diseases, thrombosis, and inflammation. DHA is commonly associated with 29 diseases, including amyotrophic lateral sclerosis, parkinsonian disorders, nerve degeneration, and dyskinesia related only to DHA. Oleylamide is associated with amnesia and seizures. Autistic disorder is related to both DHA and AA.

In relation to amphetamine, we analyzed the chemical–disease interaction association of DHA, AA, and oleylamide, in comparison with amphetamine (Figure S2). Congenital abnormalities, bradycardia, substance-related disorder, fever, hyperalgesia, and hypotension are common to AA and amphetamine. Dyskinesia, nerve degeneration, pain, liver diseases, and arrhythmias are common to DHA and amphetamine. Amnesia and seizures are common to oleylamide and amphetamine.

AA is commonly associated with cardiovascular diseases, thrombosis, and inflammation. Amyotrophic lateral sclerosis, parkinsonian disorders, and dyskinesia related only to DHA.

**Figure 2:**
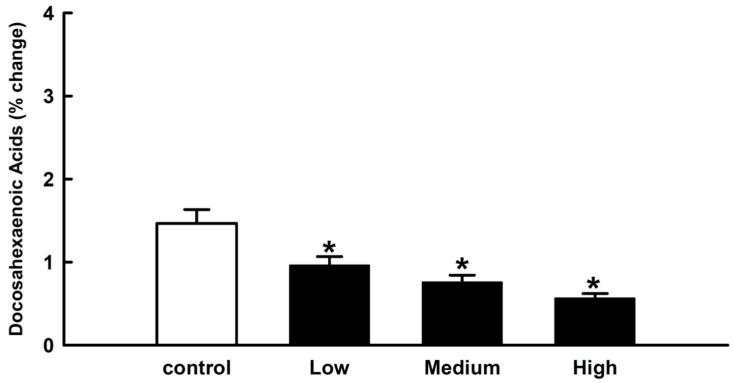
Relationship between amphetamine toxicity and levels of docosahexaenoic acid (DHA). The percentage change in the relative levels of DHA in amphetamine-related fatalities with different amphetamine concentrations (black bars) were significantly lower than the control (white bar). Values are the group mean ± SEM., * *p* ≤ 0.05 versus control.

In the current study, our focus was on DHA, hence we analyzed the relative percentage change in the level of DHA in the postmortem brain samples from amphetamine-related fatalities. The level of DHA in the brain differed significantly from that of the control group. The results also indicated that, as amphetamine levels rose, DHA levels decreased (Figure 2).

## 3. Discussion

Amphetamines disrupt biochemical processes in the body and allow several chemicals to accumulate in the brain. The number of organs affected, the dose of amphetamine taken, the presence of potent decongestants, and the patient’s preexisting medical conditions all play a role in the severity of amphetamine toxicity [22,23].

Hundreds of chemical entities have been identified in the brain tissue of fatal amphetamine cases. Many of these chemicals have not yet been explicitly studied. However, we found seven common chemicals in the three categories created, based on the amphetamine concentration in the brain. These compounds have been linked to various diseases in CTD, including neurocognitive disorders, dementia, neurodevelopmental disorders, Alzheimer’s disease, major depression, and mood disorders. CTD, a well-known database, provides manually curated information about chemical–gene/protein interactions, chemical–disease, and gene–disease relationships, and is used to better understand how chemical exposure affects human health. The generated data is combined with functional and pathway data to help develop hypotheses about the mechanisms underlying environmentally influenced diseases.

Only DHA, AA, and oleylamide were linked to curated diseases in the CTD database. Amnesia is common to both amphetamine and oleylamide, which both have a potential role in amnesia [24,25]. The findings also revealed that they are linked to seizures. In this context, amphetamine abuse causes seizures, whereas oleylamide has an anti-seizure effect [26,27]. The link between amphetamine, oleylamide, amnesia, and seizure needs to be explored. In addition, AA was identified as one of the brain chemicals in all the cases. We hypothesize that the disordered activation of lipoxygenase and cyclooxygenase during amphetamine abuse could result in various inflammatory diseases. According to Bhattacharjee et al., the acute amphetamine administration to rats results in the release of AA, a second messenger following indirect agonism of dopamine D2-like receptors in the brain [28]. This is likely due to neuroplastic changes in brain AA signaling, which correspond to depressive behaviors observed in humans and rats after chronic amphetamine withdrawal. Methamphetamine’s neurotoxicity may be due to its prostaglandin H synthase (PHS)-dependent bioactivation, which releases free radical intermediates that eventually generate reactive oxygen species and damage cellular macromolecules [29]. In response to the amphetamine challenge, DHA deficient mice had a significantly larger ventral striatum. The AA composition in the ventral striatum, as well as the AA:DHA ratio, were found to be positively correlated with DA concentrations, which were positively associated with locomotor activity [30].

According to our findings, there may be a link between an increase in amphetamine levels and a decrease in DHA levels, which could be due to amphetamine toxicity. There was no statistically significant change in AA levels due to the small sample size. It has been demonstrated that DHA deficiency may play a role in the etiology of neurological disorders. DHA and AA were frequently linked to autistic disorders, cocaine-related disorders, Alzheimer’s disease, and cognitive dysfunction. These chemicals matched with the standard curated disease list in the CTD and were considered as neurological disorders. DHA, a polyunsaturated fatty acid (FA), is selectively esterified to amino phospholipids and is abundant at the cytofacial site of the plasma membrane, where it plays a specific role in intracellular events. DHA is involved in cellular oxidative processes, intracellular signaling to gene expression, and growth regulation modulation [31]. It helps circulating blood in the brain during mental tasks [32,33], which is especially beneficial for patients with dementia and Alzheimer’s disease [34,35]. Additionally, as there is limited clinical literature, empirical evidence from preclinical studies can also be supportive. According to one study, dietary DHA deficits significantly alter mesocorticolimbic dopamine (DA) neurotransmission in rat brains [17]. Levant et al. (2004) demonstrated that rats fed a diet with a relatively minor reduction in brain DHA content exhibit adult behavioral changes consistent with altered dopaminergic function [36]. Moreover, serotonin, a neurotransmitter, may require enough DHA to function normally in the brain and is essential to stabilize mood and reduce nerve cell inflammation [30,37]. Thus, our preliminary findings suggest that amphetamine abusers may experience a deficiency of DHA which can impact cell membrane fluidity, neurotransmission, and cause neuroinflammation.

DHA deficiency has been linked to increased amphetamine-induced locomotor activity and behavioral sensitization in rodents [36,38,39]. DHA’s role in amphetamine-induced toxicity has also been demonstrated by reduced amphetamine-induced behavioral impairment, lipid peroxidation, and cytokine release in a rat treated with DHA [40]. These findings indicate that DHA deficiency contributed to amphetamine toxicity.

Other chemicals found among the groups in our study are eicosane and 2, 4 di-tert-butylphenol (DTBP). They are naturally occurring substances derived from polyunsaturated fatty acids with 20 carbons. Eicosane is effective at suppressing pro-inflammatory cytokines (TNF- and IL-12), while DTBP is cytoprotective against various oxidants. The correlation between 2,4-di-tert-butylphenol and vitamin C equivalent antioxidant capacity was strong. 2,4-di-tert-butylphenol scavenged the ABTS radical anions in a dose-dependent manner. Furthermore, in a mouse model, a 2,4-di-tert-butylphenol diet had a significant anti-amnesic effect [41]. Similarly, trace amounts of other chemicals such as 1-Octadecene, 1-tridecene, and oleylamide have also been observed in amphetamine fatal cases but did match annunciated CTD chemicals database. However, future studies would be needed to justify the down- or up-regulation of these chemicals in various tissues.

The current postmortem study’s data has limitations, including a small number of samples and varying postmortem sample intervals. Given the challenges and limitations of this study method, future studies that combine and consider details of medical history, autopsy findings, manner and cause of death, toxicological investigation, quantification of brain AA and DHA concentrations, and using a large postmortem sample size may provide a strong approach to understanding the relationship between brain fatty acid abnormalities and amphetamine toxicity in amphetamine-related fatalities.

## 4. Materials and Methods

### 4.1. Sample Population

The age group of the amphetamine-related fatal cases was 21–44 years, and the cause of death was accidental (40%), gunshot (10%), traffic accidents (10%), and suicide (40%). The age group of the control sample (non-amphetamine related fatality cases) was 20–56 years, and the cause of mortality reported was gunshot (50%) and undetermined (50%).

### 4.2. Study Design

A retrospective design was used to retrieve information on amphetamine fatalities from the Online Toxicology Analysis Requests and Results (OTARR) system, between January 2019 and December 2021. OTARR is a computerized toxicological database developed to harmonize labor policies in all the toxicological institutes of the Kingdom of Saudi Arabia. The data are confidential and not available to the public. However, access was obtained through the Ministry of Health electronic portal for health services in Saudi Arabia. All data pertaining to toxicological study results, including the cause of death in fatalities involving amphetamine, were obtained from the OTARR system. Using a data collection form, we retrieved, analyzed, and quantified fatalities associated with the use of amphetamine.

### 4.3. Data Collection

Relevant information such as age, substance of abuse (amphetamine) that led to fatality, information about non-amphetamine fatal cases (control), and brain toxicological data was extracted from the database. Amphetamine-related fatalities were classified into three groups depending on amphetamine brain concentration based on a previous investigation [5], with each group having six cases, and the fourth group, for non-amphetamine-related fatalities (control), containing six cases. The Those with less than 0.5 g/mL are classified as low concentration (low group), those with more than 0.5 g/mL but less than 1.5 g/mL as medium concentration (medium group), and those with more than 1.5 g/mL as high concentration (high group). We investigated the toxicological results and chemical profiles in amphetamine-related fatalities. We obtained information on the brain chemical profile of amphetamine-related fatalities while excluding fatalities associated with other drug abuse substances.

### 4.4. Bioinformatics Tools and Analysis

The chemical profiles were uploaded to the Comparative Toxicogenomics Database (CTD) analysis application and analyzer tools on the CTD website http://ctdbase.org/ (accessed on 30 March 2023), which identified the chemical disease association. CTD captures curated associations, which are real associations between chemicals and a disease. CTD biocurators mine published literature to extract chemical–disease associations. It reads the peer-reviewed scientific literature and manually curates three core molecular interactions between chemicals, genes, and diseases using official nomenclature, integrating third party-controlled vocabularies for chemicals, genes, diseases, and organisms, and a novel controlled vocabulary for molecular interactions. Manual curation yields a strong, densely annotated dataset with extremely accurate and detailed data. Chemical A, for example, is linked to disease B because of a curated interaction with gene C, and gene C is linked to disease B. We identified the chemicals found in all the tested samples using a Venn diagram. Only chemicals that matched and were linked to curated neurological disorders were chosen.

### 4.5. Statistical Analysis

To summarize the data, descriptive statistics were used. The results were presented as means, standard error of the mean (SEM), and median. All data were explored and calculated using SigmaPlot 11 for Windows. One-way ANOVA and Dunnett’s post hoc test were done to compare the groups. Chemical-Diseases Interaction Query http://ctdbase.org/tools/ (accessed on 30 March 2023), was used to retrieve chemical–disease relationships by selecting ‘Chemicals’ as input type, inputting a list of chemical terms that are common in all three groups, and selecting ‘Enriched Diseases’ as output. The hypergeometric distribution is used to calculate the significance of enrichment and adjusted for multiple testing using the Bonferroni method. The default values were utilized for adjusted *p*-values (threshold 0.01).

## 5. Conclusions

Amphetamine abuse can produce several chemicals which can have a derogatory impact on brain function. The altered levels of DHA, formation of AA, and oxidative products suggest that amphetamine abuse can cause neuroinflammation and neurotoxicity. Moreover, the formation of these products is linked to the development of neurological disorders, as evidenced by using the CTD tools. Although this study provides evidence, we propose to conduct further studies to substantiate the correlation between the chemicals formed and neurological disorders.

## Figures and Tables

**Table 1:** Chemicals detected in all three groups and associated enriched GO.

Chemicals	Enriched GO (Molecular Function) *	Enriched GO (Biological Process) *
1-octadecene, 1-tridecene, 2,4-di-tert-butylphenol, arachidonic acid (AA), docosahexaenoic acid (DHA), eicosane, oleylamide	Protein binding, enzyme binding, ion binding, catalytic activity, oxidoreductase activity	Cellular response to stimulus, cellular process, response to oxygen-containing compound, metabolic process, response to stress

* The significance of GO term is enriched based on corrected *p*-value threshold of 0.001.

## Data Availability

All relevant details are included in the article.

## Supplementary Materials

The supporting information can be downloaded at: https://www.mdpi.com/article/10.3390/molecules28124787/s1. Figure S1: Venn diagram showing the number of curated diseases associated with arachidonic acid (blue circle), docosahexaenoic acid (DHA) (green circle), and oleylamide (yellow circle) utilizing the CTD database; Figure S2: Venn diagram showing the number of curated diseases associated with (a) arachidonic acid (blue circle), (b) docosahexaenoic acid (DHA) (blue circle), (**c**) oleylamide (blue circle), and amphetamine (green circles) utilizing the CTD database.

## References

[1] Heal D.J., Smith S.L., Gosden J., Nutt D.J. (2013). Amphetamine, Past and Present—A Pharmacological and Clinical Perspective. J. Psychopharmacol..

[2] Åhman A., Jerkeman A., Blomé M.A., Björkman P., Håkansson A. (2018). Mortality and Causes of Death among People Who Inject Amphetamine: A Long-Term Follow-Up Cohort Study from a Needle Exchange Program in Sweden. Drug Alcohol Depend..

[3] Al-Haqwi A.I. (2010). Perception among medical students in Riyadh, Saudi Arabia, Regarding Alcohol and Substance Abuse in the Community: A Cross-Sectional Survey. Subst. Abuse Treat. Prev. Policy.

[4] Medhat B. (2013). Substance Use Disorders in Saudi Arabia: Review Article. J. Subst. Use.

[5] Attafi I.M., Tumayhi M.M., Banji D., Albeishy M.Y., Khardali I.A., Korashy H.M. (2021). Analysis of Fatalities Involving Amphetamine in Jazan, Saudi Arabia. Forensic Sci. Int. Rep..

[6] Bramness J.G., Gundersen Ø.H., Guterstam J., Rognli E.B., Konstenius M., Løberg E.-M., Medhus S., Tanum L., Franck J. (2012). Amphetamine-Induced Psychosis—A Separate Diagnostic Entity or Primary Psychosis Triggered in the Vulnerable?. BMC Psychiatry.

[7] Panenka W.J., Procyshyn R.M., Lecomte T., MacEwan G.W., Flynn S.W., Honer W.G., Barr A.M. (2013). Methamphetamine use: A Comprehensive Review of Molecular, Preclinical and Clinical Findings. Drug Alcohol Depend..

[8] Metz V.G., Segat H.J., Dias V.T., Barcelos R.C.S., Maurer L.H., Stiebe J., Emanuelli T., Burger M.E., Pase C.S. (2019). Omega-3 Decreases D1 and D2 Receptors Expression in the Prefrontal Cortex and Prevents Amphetamine-Induced Conditioned Place Preference in Rats. J. Nutr. Biochem..

[9] Calipari E.S., Ferris M.J. (2013). Amphetamine Mechanisms and Actions at the Dopamine Terminal Revisited. J. Neurosci..

[10] Faraone S.V. (2018). The Pharmacology of Amphetamine and Methylphenidate: Relevance to the Neurobiology of Attention-deficit/Hyperactivity Disorder and Other Psychiatric Comorbidities. Neurosci. Biobehav. Rev..

[11] Robinson T.E., Berridge K.C. (2000). The Psychology and Neurobiology of Addiction: An Incentive–Sensitization View. Addiction.

[12] Kula K., Rojek S., Maciów-Głąb M., Kopacz P., Kłys M. (2014). Medico-Legal Aspect of Amphetamine-Related Deaths. Arch. Med. Sądowej Kryminol./Arch. Forensic Med. Criminol..

[13] Bowyer J.F., Hanig J.P. (2014). Amphetamine-and Methamphetamine-Induced Hyperthermia: Implications of the Effects Produced in Brain Vasculature and Peripheral Organs to Forebrain Neurotoxicity. Temperature.

[14] Song Y.W., Lim Y., Cho S.K. (2018). 2, 4-Di-Tert-Butylphenol, a Potential HDAC6 Inhibitor, Induces Senescence and Mitotic Catastrophe in Human Gastric Adenocarcinoma AGS cells. Biochim. Biophys. Acta (BBA) Mol. Cell Res..

[15] Degenhardt L., Baxter A.J., Lee Y.Y., Hall W., Sara G.E., Johns N., Flaxman A., Whiteford H.A., Vos T. (2014). The Global Epidemiology and Burden of Psychostimulant Dependence: Findings from the Global Burden of Disease Study 2010. Drug Alcohol Depend..

[16] Bazinet R.P., Layé S. (2014). Polyunsaturated Fatty Acids and Their Metabolites in Brain Function and Disease. Nat. Rev. Neurosci..

[17] McNamara R.K., Hahn C.-G., Jandacek R., Rider T., Tso P., Stanford K.E., Richtand N.M. (2007). Selective Deficits in the Omega-3 Fatty Acid Docosahexaenoic Acid in the Postmortem Orbitofrontal Cortex of Patients with Major Depressive Disorder. Biol. Psychiatry.

[18] McNamara R.K., Jandacek R., Rider T., Tso P., Stanford K.E., Hahn C.-G., Richtand N.M. (2008). Deficits in Docosahexaenoic Acid and Associated Elevations in the Metabolism of Arachidonic Acid and Saturated Fatty Acids in the Postmortem Orbitofrontal Cortex of Patients with Bipolar Disorder. Psychiatry Res..

[19] Schneider M., Levant B., Reichel M., Gulbins E., Kornhuber J., Müller C.P. (2017). Lipids in Psychiatric Disorders and Preventive Medicine. Neurosci. Biobehav. Rev..

[20] Hamazaki K., Hamazaki T., Inadera H. (2012). Fatty Acid Composition in the Postmortem Amygdala of Patients with Schizophrenia, Bipolar Disorder, and Major Depressive Disorder. J. Psychiatr. Res..

[21] Marcheselli V.L., Hong S., Lukiw W.J., Tian X.H., Gronert K., Musto A., Hardy M., Gimenez J.M., Chiang N., Serhan C.N. (2003). Novel Docosanoids Inhibit Brain Ischemia-Reperfusion-Mediated Leukocyte Infiltration and Pro-Inflammatory Gene Expression. J. Biol. Chem..

[22] Berman S., O’Neill J., Fears S., Bartzokis G., London E.D. (2008). Abuse of Amphetamines and Structural Abnormalities in the Brain. Ann. N. Y. Acad. Sci..

[23] Vasan S., Olango G.J. (2022). Amphetamine Toxicity. StatPearls.

[24] Heo H.-J., Park Y.-J., Suh Y.-M., Choi S.-J., Kim M.-J., Cho H.-Y., Chang Y.-J., Hong B., Kim H.-K., Kim E. (2003). Effects of Oleamide on Choline Acetyltransferase and Cognitive Activities. Biosci. Biotechnol. Biochem..

[25] Gibbs M.E., Ng K.T. (1977). Counteractive Effects of Norepinephrine and Amphetamine on Ouabain-Induced Amnesia. Pharmacol. Biochem. Behav..

[26] Alldredge B.K., Lowenstein D.H., Simon R.P. (1989). Seizures Associated with Recreational Drug Abuse. Neurology.

[27] Wu C.-F., Li C.-L., Song H.-R., Zhang H.-F., Yang J.-Y., Wang Y.-L. (2003). Selective Effect of Oleamide, an Endogenous Sleepinducing Lipid Amide, on Pentylenetetrazole-Induced Seizures in Mice. J. Pharm. Pharmacol..

[28] Bhattacharjee A.K., Chang L., Chen M., White L., Bell J.M., Bazinet R.P., Rapoport S.I. (2008). Chronic d-Amphetamine Depresses an Imaging Marker of Arachidonic Acid Metabolism in Rat Brain. Int. J. Neuropsychopharmacol..

[29] Ramkissoon A., Wells P.G. (2011). Human Prostaglandin H synthase (hPHS)-1 and hPHS-2 in Amphetamine Analog Bioactivation, DNA Oxidation, and Cytotoxicity. Toxicol. Sci..

[30] McNamara R.K. (2016). Role of Omega-3 Fatty Acids in the Etiology, Treatment, and Prevention of Depression: Current Status and Future Directions. J. Nutr. Intermed. Metab..

[31] Yavin E. (2006). Versatile Roles of Docosahexaenoic Acid in the Prenatal Brain: From Pro-and Anti-Oxidant Features to Regulation of Gene Expression. Prostaglandins Leukot. Essent. Fat. Acids.

[32] Jackson P.A., Reay J.L., Scholey A.B., Kennedy D.O. (2012). DHA-Rich Oil Modulates the Cerebral Haemodynamic Response to Cognitive Tasks in Healthy Young Adults: A near IR Spectroscopy Pilot Study. Br. J. Nutr..

[33] Jackson P.A., Forster J.S., Bell J.G., Dick J.R., Younger I., Kennedy D.O. (2016). DHA Supplementation alone or in Combination with Other Nutrients does not Modulate Cerebral Hemodynamics or Cognitive Function in Healthy Older Adults. Nutrients.

[34] Yassine H.N., Braskie M.N., Mack W.J., Castor K.J., Fonteh A.N., Schneider L.S., Harrington M.G., Chui H.C. (2017). Association of Docosahexaenoic Acid Supplementation with Alzheimer Disease Stage in Apolipoprotein E ε4 Carriers: A review. JAMA Neurol..

[35] Yanai H. (2017). Effects of N-3 Polyunsaturated Fatty Acids on Dementia. J. Clin. Med. Res..

[36] Levant B., Radel J.D., Carlson S.E. (2004). Decreased Brain Docosahexaenoic Acid during Development Alters Dopamine-Related Behaviors in Adult Rats That Are Differentially Affected by Dietary Remediation. Behav. Brain Res..

[37] Bozzatello P., Brignolo E., De Grandi E., Bellino S. (2016). Supplementation with Omega-3 Fatty Acids in Psychiatric Disorders: A Review of Literature Data. J. Clin. Med..

[38] McNamara R.K., Sullivan J., Richtand N.M., Jandacek R., Rider T., Tso P., Campbell N., Lipton J. (2008). Omega-3 Fatty Acid Deficiency Augments Amphetamine-Induced Behavioral Sensitization in Adult DBA/2J Mice: Relationship with Ventral Striatum Dopamine Concentrations. Synapse.

[39] McNamara R.K., Sullivan J., Richtand N.M. (2008). Omega-3 Fatty Acid Deficiency Augments Amphetamine-Induced Behavioral Sensitization in Adult Mice: Prevention by Chronic Lithium Treatment. J. Psychiatr. Res..

[40] El-Sisi A.E.-S., Sokkar S.S., El-Sayad M.E.-S., Ramadan E.S., Osman E.Y. (2016). Celecoxib and Omega-3 Fatty Acids alone and in Combination with Risperidone Affect the Behavior and Brain Biochemistry in Amphetamine-Induced Model of Schizophrenia. Biomed. Pharmacother..

[41] Choi S.J., Kim J.K., Kim H.K., Harris K., Kim C.-J., Park G.G., Park C.-S., Shin D.-H. (2013). 2, 4-Di-Tert-Butylphenol from Sweet Potato Protects against Oxidative Stress in PC12 Cells and in Mice. J. Med. Food.

