# Supplementary material for: Amphetamine-Related Fatalities and Altered Brain Chemicals: A Preliminary Investigation Using the Comparative Toxicogenomic Database
Source: Molecules. 2023 Jun 15;28(12):4787. doi: 10.3390/molecules28124787 (PMC10305267; doi:10.3390/molecules28124787)
Supplement: Supplementary file 1 [file molecules-28-04787-s001.zip › molecules-2405655-supplementary.pdf]

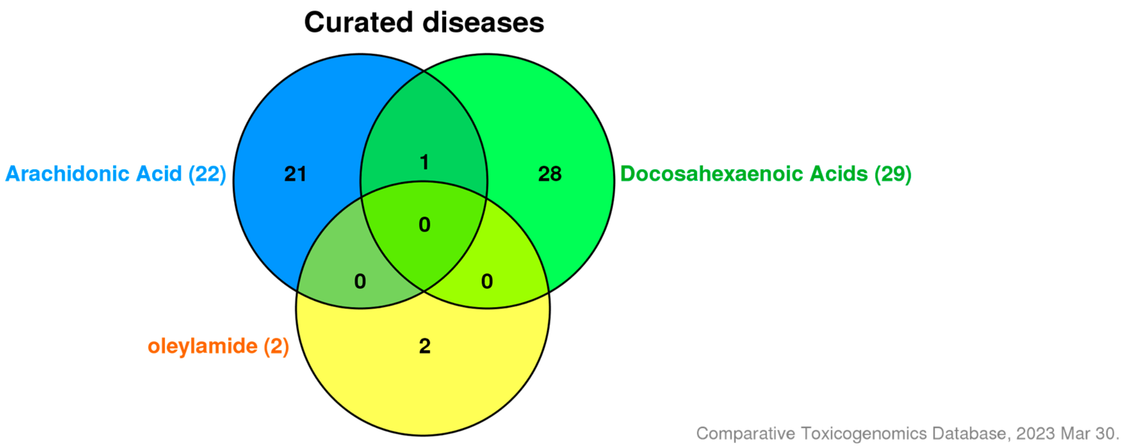

Figure S1. Venn diagram show the number of curated diseases associated with arachidonic acid (blue circle), docosahexaenoic acid (DHA) (green circle), and oleylamide (yellow circle) utilizing the CTD database.

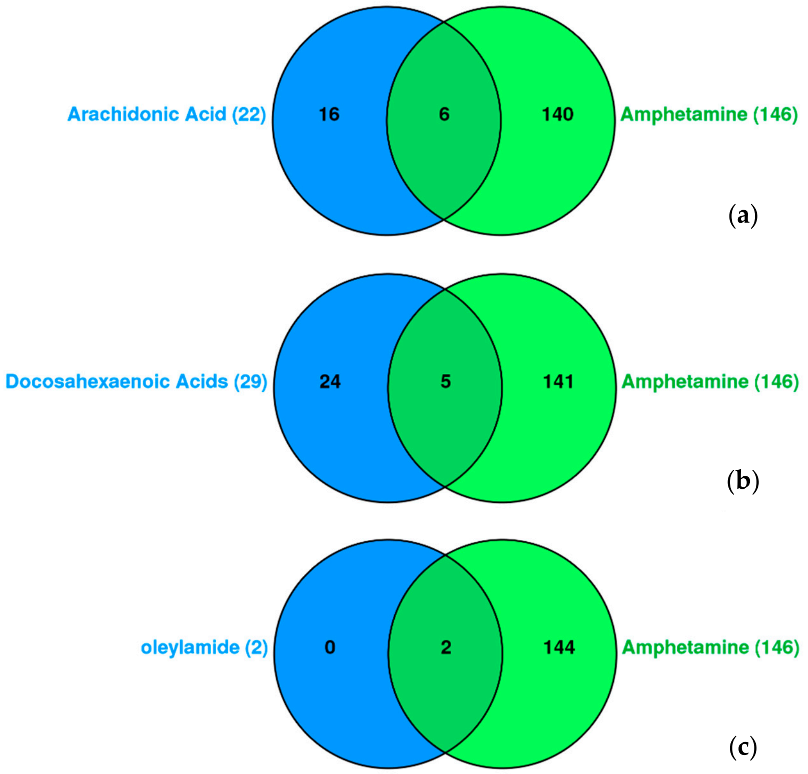

Figure S2. Venn diagram show the number of curated diseases associated with (**a**) arachidonic acid (blue circle), (**b**) docosahexaenoic acid (DHA) (blue circle), (**c**) oleylamide (blue circle), and amphetamine (green circles) utilizing the CTD database.
